# Cardiovascular correlates of epigenetic aging across the adult lifespan: a population-based study

**DOI:** 10.1007/s11357-022-00714-0

**Published:** 2023-02-08

**Authors:** Dan Liu, N. Ahmad Aziz, Gökhan Pehlivan, Monique M. B. Breteler

**Affiliations:** 1 German Center for Neurodegenerative Diseases (DZNE), Population Health Sciences, Venusberg-Campus 1, Building 99, 53127 Bonn, Germany; 2 Department of Neurology, Faculty of Medicine, University of Bonn, Bonn, Germany; 3 Institute for Medical Biometry, Informatics and Epidemiology (IMBIE), Faculty of Medicine, University of Bonn, Bonn, Germany

**Keywords:** Biological age, Epigenetic age acceleration, Cardiovascular aging

## Abstract

Individuals with a similar chronological age can exhibit marked differences in cardiovascular risk profiles, but it is unknown whether this variation is related to different rates of biological aging. Therefore, we investigated the relation between nine domains of cardiovascular function and four epigenetic age acceleration estimators (i.e., AgeAccel.Horvath, AgeAccel.Hannum, AgeAccelPheno, and AgeAccelGrim), derived from DNA methylation profiles. Among 4194 participants (mean age 54.2 years (range 30.0–95.0)) from the Rhineland Study, an ongoing population-based cohort study in Bonn, Germany, epigenetic age acceleration increased by 0.19–1.84 years per standard deviation (SD) increase in cardiovascular risk across multiple domains, including measures of kidney function, adiposity, and a composite cardiovascular risk score. Measures of inflammation and glucose homeostasis were associated with AgeAccel.Hannum, AgeAccelPheno, and AgeAccelGrim, but not with AgeAccel.Horvath. Moreover, effect sizes were larger for AgeAccelPheno and AgeAccelGrim than for AgeAccel.Horvath and AgeAccel.Hannum. Similarly, epigenetic age acceleration increased by 0.15–0.81 years per SD increase in markers of vascular function (blood pressure, arterial stiffness, and hemodynamic measures), whereas better endothelial function was only associated with lower AgeAccelGrim. Most effects on epigenetic age acceleration were independent, which suggests they independently contribute to different rates of biological aging.

**Supplementary Information:**

The online version contains supplementary material available at 10.1007/s11357-022-00714-0.

## Introduction

Cardiovascular diseases (CVDs) are the leading causes of morbidity and mortality worldwide [1, 2]. However, substantial inter-individual variation in cardiovascular aging and associated morbidity remains in individuals with the same chronological age, pointing towards markedly different rates of biological aging [3–7]. Nevertheless, the extent to which different cardiovascular factors contribute to biological aging is still unclear. Elucidation of the relation between inter-individual differences in cardiovascular factors and the rate of biological aging is crucial for the development of more sensitive and specific surrogate biomarkers of CVDs, which could facilitate the development of preventive and therapeutic strategies for CVDs based on promoting healthy aging approaches.

DNA methylation is a major form of epigenetic modulation that is critically involved in the regulation of gene expression. With increasing age, the methylation status of numerous DNA cytosine-phosphate-guanine [8] sites differentially changes across the genome, reflecting the effects of cumulative exposure to major risk factors involved in the pathogenesis of age-related conditions [9–11]. Indeed, several studies have shown that classical cardiovascular risk factors causally affect methylation status [12–18]. Large scale epigenomic analyses have shown that body mass index drives differential methylation status in the blood, adipose tissue, and liver, as well as changes in methylation over time [13, 14, 16, 18]. Inter-individual variation in blood lipids, as well as hyperglycemia, could induce differential methylation changes in blood cells, human endothelial cells and skeletal muscle [12, 15, 17]. In addition, data from in vitro studies suggest that blood flow induced methylation modifications are related to endothelial and vascular functions, which may lead to atherosclerosis and cardiovascular diseases [19, 20]. The latter is also supported by a recent population-based epigenetic study, which showed a bidirectional association between blood pressure and DNA methylation [21]. However, the association of quantitative markers of vascular function, including age-related changes in arterial stiffness, endothelial function, and hemodynamics, with DNA methylation remains largely unknown.

DNA methylation profiles have been used to estimate biological age, serving as so called epigenetic clocks. First-generation epigenetic clocks, including Horvath’s and Hannum’s clocks, were developed using chronological age as a surrogate for biological age [22, 23]. However, it is crucial to not only include CpGs that display changes with chronological time, but also those that account for substantial variation in physiological and molecular characteristics among individuals of the same chronological age. Thus, second-generation epigenetic clocks, including phenotypic age (PhenoAge) and GrimAge were optimized to capture multi-system physiological dysfunctions and health span [24, 25]. PhenoAge, trained on mortality-related clinical biomarkers, and GrimAge, developed using plasma proteins that are associated with age-related conditions, more closely reflect the high inter-individual variability in the underlying biological aging processes than the first-generation epigenetic clocks. Moreover, transcriptional analysis also revealed that genes linked to Horvath/Hannum’s clocks are only related to development and differentiation pathways [26]. By contrast, genes associated with PhenoAge and GrimAge are involved in crucial aging pathways, including increased activation of pro-inflammatory and interferon pathways, cytokine-mediated signaling pathway, mitochondrial signatures, and fatty acid transmembrane transport [24, 25]. Previous studies have shown that the discrepancy between an individual’s epigenetic age and chronological age, referred to as epigenetic age acceleration (including AgeAccel.Horvath, AgeAccel.Hannum, AgeAccelPheno, and AgeAccelGrim), is associated with age-related phenotypes and is a strong predictor of all-cause as well as cardiovascular mortality [26–32]. Recent studies further showed that AgeAccelGrim outperforms other epigenetic age acceleration estimators in the prediction of age-related diseases and mortality [33, 34].

An important question remains whether and how inter-individual differences in cardiovascular risk factor profiles (including levels of lipoproteins and measures of kidney function, inflammation, adiposity, and glucose homeostasis), as well as changes in makers of vascular function (blood pressure, arterial stiffness, endothelial function, hemodynamics), are associated with epigenetic age acceleration at the population-level. In particular, the relation between cardiovascular risk factors and AgeAccelPheno and AgeAccelGrim has received little study [35–37]. Moreover, the association of quantitative and highly sensitive markers of vascular function with these four epigenetic estimators has not been investigated, and therefore, the comparative utility of the first- and second-generation epigenetic age acceleration estimators in capturing similarities and differences in multi-domain cardiovascular dysfunction remains to be established.

We aimed to investigate whether a comprehensive set of cardiovascular risk factors involving multiple domains, as well as quantitative markers of vascular function, have consistent and independent effects on four epigenetic age acceleration estimators across a wide age range in the general population. We postulated that individuals with unfavorable profiles of cardiovascular risk factors and quantitative markers of vascular function would exhibit higher epigenetic age acceleration, and that lifespan estimators (i.e., AgeAccelPheno and AgeAccelGrim) would outperform the first-generation epigenetic clocks (i.e., AgeAccel.Horvath, AgeAccel.Hannum).

## Methods

### Study population

This study was based on the Rhineland Study, an ongoing single-center, population-based cohort study that recruits people aged 30 years and above from 2 geographically defined areas in Bonn, Germany. The only exclusion criterion is the insufficient command of the German language to provide informed consent. Persons living in the recruitment areas are predominantly German from Caucasian descent. A primary objective of the Rhineland Study is to identify determinants and markers of healthy aging. For this, we use a deep-phenotyping approach. At baseline, participants complete an 8-h in-depth multi-domain phenotypic assessment. Moreover, various types of biomaterials, including blood samples (buffy coat, serum, EDTA-plasma), urine, stool, and hair samples, are collected. Approval to undertake the study was obtained from the ethics committee of the University of Bonn, Medical Faculty. We obtained written informed consent from all participants in accordance with the Declaration of Helsinki.

As the recruitment in the Rhineland Study is ongoing, for the current analyses, we used baseline data of the first 4200 consecutively participants of the Rhineland Study with methylation data. We excluded samples that did not meet the methylation data quality control criteria (*n* = 6). The final analysis sample comprised 4194 participants.

### DNA methylation quantification

Genomic DNA was extracted from buffy coat fractions of anti-coagulated blood samples using Chemagic DNA buffy coat kit (PerkinElmer, Germany) with Chemagic Magnatic Separation Module 1 and Chemagic Prime 8 Automated Workstation, and was subsequently bisulfite converted using the EZ-96DNA Methylation-Lightning^TM^MagPrep from Zymo according to the manufacturer’s instructions. DNA methylation levels were measured on Illumina iScan using Illumina’s Human MethylationEPIC BeadChip. The methylation level for each probe was derived as a beta value representing the fractional level of DNA methylation at that probe. Sample-level and probe-level quality control was performed using the ‘minfi’ package [38] in R (version 3.5.0). Samples with sex mismatch or a missing rate at > 1% across all probes were excluded. Probes with a missing rate > 1% (at a detection *p* value > 0.01) were also excluded following previously published recommendation guidelines for analyzing methylation data [39].

### Estimation of epigenetic age acceleration

Four epigenetic clocks were utilized: scores on Horvath and Hannum’s clocks were calculated according to the algorithms described by Horvath et al. and Hannum et al., using 353 and 71 CpG sites, respectively [22, 23]. PhenoAge and GrimAge were calculated based on the algorithms developed by Levine et al. and Lu et al., using 513 and 1030 CpG sites, respectively [24, 25]. Epigenetic age acceleration is defined as the residual (in years) that results from regressing epigenetic age on chronological age. The corresponding age-adjusted measures of epigenetic age acceleration are denoted as AgeAccel.Horvath, AgeAccel.Hannum, AgeAccelPheno, and AgeAccelGrim.

### Measurement of classical cardiovascular risk factors

Blood samples were collected between 7:00 and 9:45 in the morning from an antecubital or dorsal hand vein after overnight fasting. Low-density lipoprotein (LDL) cholesterol, high-density lipoprotein (HDL) cholesterol, triglycerides, total cholesterol, cystatin C, C-reactive protein (CRP), insulin, glucose, and glycated hemoglobin (HbA1c) concentrations in venous blood samples were measured using standard methods at the local clinical chemistry laboratory of the University Hospital of Bonn. Insulin resistance was calculated as: insulin (mIU/L) × glucose (mmol/L)/22.5. Estimated glomerular filtration rate (eGFR) was estimated using the CKD-EPI equation [40]. Percentage of body fat was measured by direct segmental multi-frequency bioelectrical impedance analysis (InBody770). Body mass index (BMI) was calculated as weight in kilograms divided by height in meters squared. Waist circumference [41] was measured according to WHO recommendations, localizing the middle anatomical point between the lowest rib and the iliac crest with an anthropometric tape (SECA 201). The Framingham 10-year cardiovascular risk score was calculated for individuals from 30 to 79 years old without coronary heart disease, stroke, and peripheral arterial diseases, using published gender-specific algorithms [42].

### Measurement of blood pressure

Systolic blood pressure (SBP) and diastolic blood pressure (DBP) were measured three times (separated by 10 min intervals), using an oscillometric blood pressure device (Omron 705 IT). The measurements were performed while people were sitting in a resting chair in a quiet environment, and the average of the second and third measurements was used for further calculation. Mean arterial pressure [43] is calculated as (SBP + 2 × DBP)/3. Pulse pressure (PP) is the difference between SBP and DBP.

### Measurement of arterial stiffness

Arterial stiffness was assessed by total arterial compliance index (TACI, mL/mmHg/m^2^), aorta-femoral pulse wave velocity (PWV, m/s) and ankle-brachial index (ABI). TACI was calculated as stroke volume (SV, mL) divided by PP and then multiplied with body surface area (BSA, m^2^). PWV was assessed with an integrated oscillometric method, defining the propagation time of the pulse wave as the delay between opening of the aortic valve determined with impedance cardiography (ICG) waves and the arrival of the pulse wave to the mid-femoral cuff. PWV was calculated as the distance measured between the supra-sternal notch and the mid-femoral cuff divided by propagation time. ABI, calculated as the ratio of the ipsilateral ankle and brachial SBP, was measured on both sides with oscillometry. In cases where the ABI on both sides was lower than 1.40, the lower value was used for analysis, whereas in other cases the higher value was used as recommended previously [44].

### Measurement of endothelial function

Endothelial function was assessed as reactive skin hyperemia (RSH) with a laser Doppler flowmetry device (Moors, UK) using a local thermal heating protocol. Skin blood flow (SBF) on the ventral surface of the forearm was measured for a total of 26 min. After 2 min of baseline SBF measurement, the area was heated up to 40 °C until the end of the examination. The baseline SBF is followed by a nadir, and after approximately 20 min, it reaches a plateau that is linked to nitric oxide production capacity of the endothelial cells [45]. RSH was calculated as ([(plateau SBF – baseline SBF)/baseline SBF)] × 100).

### Measurement of hemodynamics

Hemodynamics was assessed by cardiac index (CI, L/min/m^2^), systemic vascular resistance index (SVRI, dynes/s/cm^5^/m^2^) and stroke index (SI, mL/m^2^). They were measured beat-to-beat for approximately 8 min with an impedance cardiography device (CardioScreen 2000, Medis, Germany) and computed by Cardiovascular Lab Software (Medis, Germany based on stoke volume (SV, mL). Briefly, cardiac output (CO [L/min]) was computed as SV multiplied by heart rate [beat per minute]; CI was computed as CO divided by BSA. SVRI was calculated as MAP divided by CO, multiplied by 80. SI was computed as SV divided by BSA.

### Demographic and health variables

We included age, sex, and education level as demographic covariates. Education level was grouped as less than high school, high school, or higher. Smoking status was defined as “current smoker” or “non-current smoker” based on self-report: current smokers were defined as those who reported smoking within a year from the examination date. Non-current smokers were defined as those who had not smoked in the lifetime or those who had quit smoking more than a year before the examination date. Missing smoking values were imputed based on cotinine metabolite levels: individuals with a cotinine level exceeding the non-current smoker sample-defined 97.5 percentile were classified as smokers. Participants were considered to have diabetes if they had a self-reported physician diagnosis of diabetes, glycated hemoglobin (%) levels of 6.5% or more, or used anti-diabetic medication, as defined according to the anatomical therapeutic chemical (ATC) code A10, which were used regularly (i.e., daily, every other day, weekly) during the past year. Hypertension was defined as a self-reported physician diagnosis of hypertension, or an average systolic blood pressure ≥ 140 mmHg and/or diastolic blood pressure ≥ 90 mmHg, or the use of antihypertensive drugs (ATC code C02, C03, C07, C08, C09) which were used regularly (i.e., daily, every other day, weekly) during the past year. Stroke and myocardial infarction were defined as a self-reported physician diagnosis.

### Statistical analysis

Data were summarized as mean ± standard deviation (SD) or counts with proportions, for continuous and categorical variables, respectively. Differences between women and men were compared using linear regression for continuous variables, and logistic regression for categorical variables, adjusting for age. Pearson correlation coefficients were used to assess correlations among cardiovascular factors, and epigenetic age acceleration. Moreover, we used hierarchical clustering to group cardiovascular risk factors, as well as markers of vascular function into homogenous clusters according to their degree of interrelatedness (R package ‘ClustOfVar’).

We assessed the relation between each cardiovascular factor (independent variable) and each epigenetic age acceleration estimator (dependent variable) using multiple linear regression. All cardiovascular variables were standardized before further analyses in order to enable better comparison of the effect sizes across different physiological domains. For all the analyses, complete data were used. In the first model, we adjusted for batch information. In model 2, we additionally adjusted for sex, which is more meaningful when it comes to AgeAccel.Horvath, AgeAccel.Hannum, and AgeAccelPheno, as GrimAge already takes sex effect into account by using sex as a covariate in its definition [25]. Model 3 was further adjusted for smoking status (current or not). As AgeAccelGrim is a composite biomarker derived from DNAm-based surrogate biomarkers of seven plasma protein levels — i.e., adrenomedullin (ADM), beta-2 microglobulin (B2M), cystatin C, growth differentiation factor 15 (GDF-15), leptin, plasminogen activation inhibitor 1 (PAI-1), tissue inhibitor metalloproteinase 1 (TIMP-1)-, and smoking pack-years (PACKYRS) [25], we further assessed the association between cardiovascular factors and the 8 corresponding surrogate variables (i.e., DNAmADM, DNAmB2M, DNAmCystatinC, DNAmGDF15, DNAmLeptin, DNAmPAI1, DNAmTIMP1, DNAmPACKYRS) to explore which underlying DNAm-based biomarkers drive the associations. To correct for multiple comparisons, we used Bonferroni correction to account for nine independent cardiovascular domains derived from hierarchical clustering, considering *P* < 0.0056 (0.05/9) as statistically significant.

As parameters within each physiologic cluster were highly correlated, we additionally calculated an average *Z* score for each cluster. We then included these cluster scores in one multivariable regression model to assess the independent relation of each cluster with epigenetic age acceleration, adjusting for sex, batch information, and smoking status.

To explore sex differences between cardiovascular factors and epigenetic age acceleration, we assessed the interaction between sex and each cardiovascular factor and performed sex-stratified analyses, if the interaction term was statistically significant. To test whether our results would be affected by cardiovascular co-morbidity, we also performed a sensitivity analysis by excluding participants with diabetes, stroke, or myocardial infarction. Potential nonlinear relationships were examined by plotting each cardiovascular factor against epigenetic age acceleration. If there was a potentially nonlinear relationship based on visual inspection, quadratic terms for cardiovascular factor were added to the regression models. All standardized effect estimates are reported with their 95% confidence intervals (CIs).

## Results

The characteristics of the study population are presented in Table 1. A total of 4194 participants had DNA methylation data available and were included in the analyses.

**Table 1:** Characteristics of the study population

	Overall (*n* = 4194)	Women (*n* = 2280)	Men (*n* = 1914)	*Adjusted p* value*
Demographic characteristics				
Age, year				0.523
Mean (SD)	54.2 (13.6)	54.0 (13.3)	54.3 (13.9)	
Median [Min, Max]	54.0 [30.0, 95.0]	54.0 [30.0, 95.0]	54.0 [30.0, 90.0]	
Education, *n* (%)				< 0.001
Low	106 (2.5%)	74 (3.2%)	32 (1.6%)	
Middle	1819 (43.4%)	1106 (48.6%)	713 (37.3%)	
High	2269 (54.1%)	1100 (48.2%)	1169 (61.1%)	
Current smoking, *n* (%)	545 (13.0%)	284 (12.5%)	261 (13.6%)	0.078
Hypertension, *n* (%)	1456 (34.7%)	696 (30.5%)	760 (39.8%)	< 0.001
Diabetes, *n* (%)	178 (4.2%)	68 (3.0%)	110 (5.7%)	< 0.001
Stroke, *n* (%)	61 (1.5%)	29 (1.3%)	32 (1.7%)	0.155
Myocardial infarction, *n* (%)	60 (1.4%)	16 (0.7%)	44 (2.3%)	< 0.001
Epigenetic age acceleration, year, mean (SD)				
AgeAccel.Horvath	0.3 (5.3)	− 0.3 (5.1)	1.0 (5.4)	< 0.001
AgeAccel.Hannum	0.3 (5.6)	− 0.6 (5.7)	1.4 (5.4)	< 0.001
AgeAccelPheno	0.1 (6.6)	− 0.4 (6.7)	0.7 (6.4)	< 0.001
AgeAccelGrim	− 0.2 (7.3)	− 1.1 (7.1)	0.9 (7.4)	< 0.001
Cardiovascular factors, mean (SD)				
LDL, mg/dL	127 (35.5)	126 (36.6)	128 (34.1)	0.171
HDL, mg/dL	62.2 (17.7)	69.6 (17.1)	53.3 (13.8)	< 0.001
LDL/HDL ratio, %	2.2 (0.9)	1.9 (0.8)	2.6 (0.9)	< 0.001
Triglyceride, mg/dL	111 (67.6)	96.3 (49.7)	128 (80.7)	< 0.001
Total cholesterol, mg/dL	199 (38.9)	203 (39.6)	194 (37.6)	< 0.001
Cystatin C, mg/L	0.9 (0.2)	0.9 (0.2)	0.9 (0.2)	< 0.001
Estimated glomerular filtration rate, ml/min/1.73m^2^	91.6 (18.3)	91.4 (17.5)	91.7 (19.3)	0.238
C-reactive protein, mg/L	1.8 (3.2)	1.8 (3.3)	1.7 (3.0)	0.261
Percentage of body fat, %	27.9 (9.0)	31.8 (8.5)	23.3 (7.1)	< 0.001
BMI, kg/m^2^	25.7 (4.2)	25.2 (4.5)	26.4 (3.7)	< 0.001
Waist circumference, cm	87.6 (12.9)	82.0 (11.5)	94.3 (11.2)	< 0.001
Insulin, mIU/L	10.3 (7.5)	9.3 (5.9)	11.5 (8.9)	< 0.001
Insulin resistance	2.4 (2.3)	2.1 (1.8)	2.8 (2.7)	< 0.001
Glucose, mg/dL	92.0 (15.3)	89.5 (14.6)	95.0 (15.5)	< 0.001
HbA1c, mmol/mol	36.0 (5.5)	35.9 (5.2)	36.2 (5.9)	0.010
Cardiovascular risk score^#^	0.4 (0.4)	0.1 (0.1)	0.8 (0.2)	< 0.001
SBP, mmHg, mean (SD)	126 (15.7)	123 (16.4)	130 (13.8)	< 0.001
DBP, mmHg, mean (SD)	75.5 (9.3)	73.8 (9.1)	77.4 (9.2)	< 0.001
Mean arterial pressure, mmHg	92.3 (10.5)	90.0 (10.6)	95.0 (9.8)	< 0.001
Pulse pressure, mmHg	51.5 (10.4)	50.2 (10.8)	53.0 (9.6)	< 0.001
Total arterial compliance index, mL/mmHg/m^2^	1.1 (0.3)	1.1 (0.3)	1.0 (0.2)	< 0.001
Pulse wave velocity, m/s	6.8 (1.4)	6.6 (1.4)	6.9 (1.4)	< 0.001
Ankle-Brachial index	1.2 (0.1)	1.1 (0.1)	1.2 (0.1)	0.002
Log reactive skin hyperemia, log (%)	5.8 (0.9)	5.8 (0.9)	5.8 (0.9)	0.143
Cardiac index, L/min/m^2^	3.2 (0.5)	3.3 (0.5)	3.0 (0.5)	< 0.001
Systemic vascular resistance index, dynes s/cm^5^/m^2^	2120 (469)	1970 (409)	2300 (474)	< 0.001
Stroke index, mL/m^2^	52.1 (8.7)	53.7 (8.7)	50.2 (8.3)	< 0.001

*HDL*, high-density lipoproteins; *LDL*, low-density lipoproteins; *BMI*, body mass index; *HbA1c*, glycated hemoglobin, *SBP*, systolic blood pressure; *DBP*, diastolic blood pressure; *SD*, standard deviation

^#^Cardiovascular risk score was calculated among participants < 80 years old without cardiovascular diseases (*n* = 3982)

^*^Comparison between women and men, adjusted for age

### Estimations of epigenetic age acceleration

AgeAccel.Horvath, AgeAccel.Hannum, and AgeAccelPheno were all moderately correlated with each other (*r* = 0.59–0.61). AgeAccelGrim was weakly correlated with AgeAccelPheno (*r* = 0.32) and the first-generation estimators (*r* = 0.16–0.21). Epigenetic age acceleration was significantly higher in men than women for all the four measures (Table 1).

### Relation between classical cardiovascular risk factors and epigenetic age acceleration

**Fig. 1:**
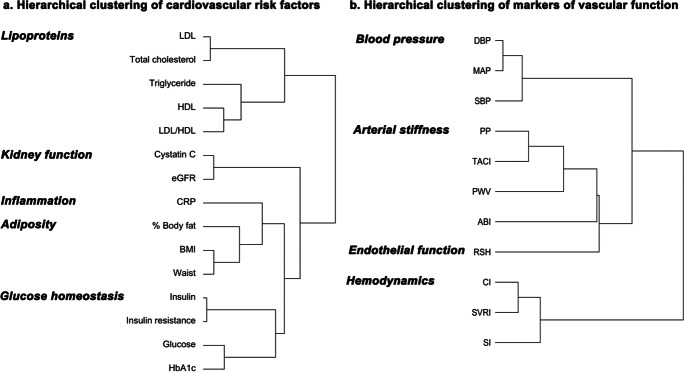
Hierarchical clustering of cardiovascular factors. Abbreviations: LDL, low-density lipoproteins; HDL, high-density lipoproteins; eGFR, estimated glomerular filtration rate; CRP, C-reactive protein; BMI, body mass index; HbA1c, glycated hemoglobin; DBP, diastolic blood pressure; MAP, mean arterial pressure; SBP, systolic blood pressure; PP, pulse pressure; TACI, total arterial compliance index; PWV, pulse wave velocity; ABI, ankle-brachial index; RSH, reactive skin hyperemia; CI, cardiac index; SVRI, systemic vascular resistance index; SI, stroke index

Conforming to their known physiological interrelations, hierarchical clustering of the classical cardiovascular risk factors yielded five categories (Fig. 1a), comprising of lipoproteins (LDL, total cholesterol, triglyceride, HDL, LDL/HDL ratio), kidney function (cystatin C and eGFR), inflammation (CRP), adiposity (% body fat, BMI, waist circumference), and glucose homeostasis (blood insulin levels, insulin resistance, blood glucose levels, HbA1c). Most of these classical cardiovascular risk factors and risk factor categories were only weakly correlated with each other (eFig. 1), indicating that different risk factor categories indeed represent different physiological domains.

**Fig. 2:**
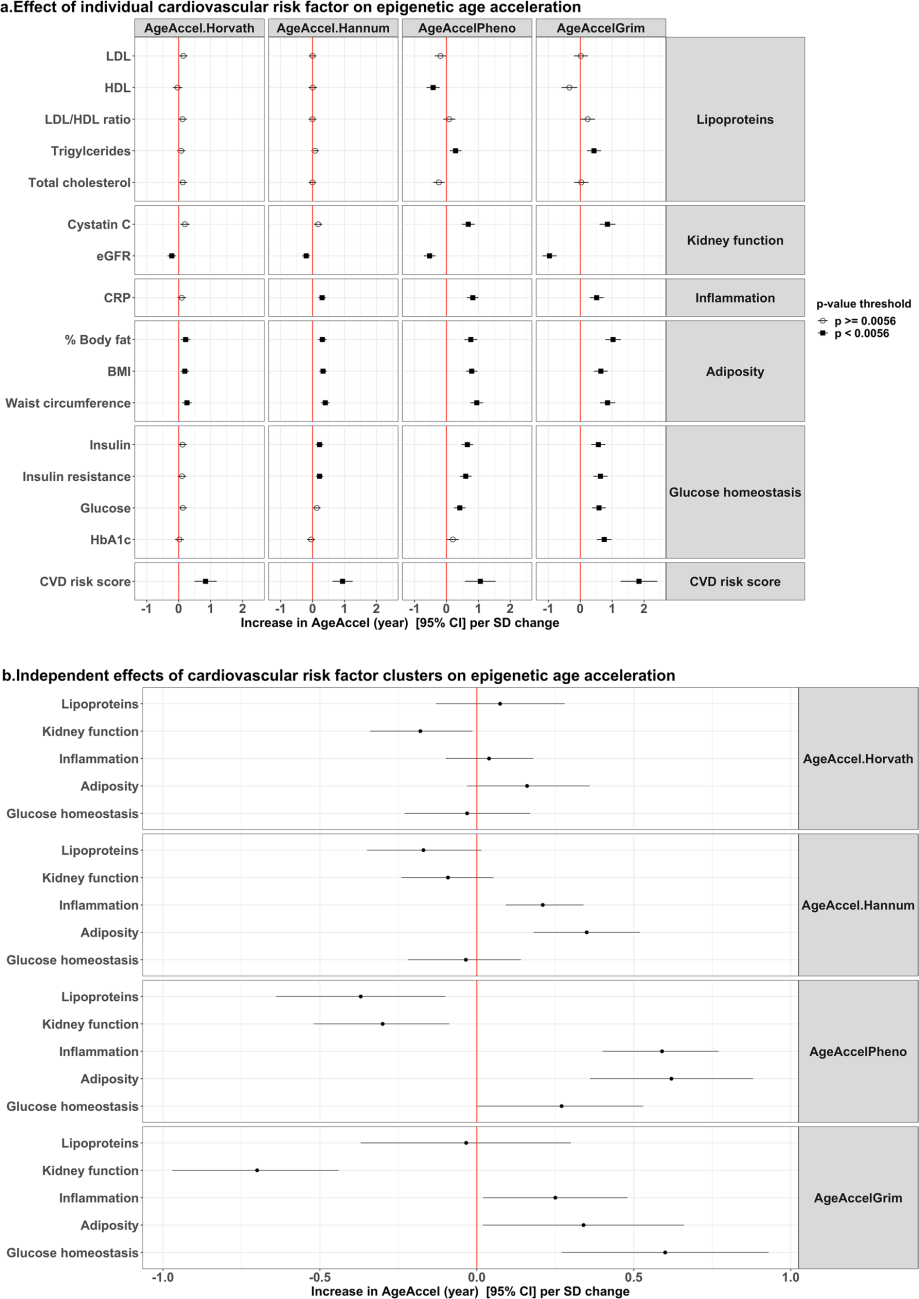
Relation between classical cardiovascular risk factors and epigenetic age acceleration. **a** Effect of individual cardiovascular risk factors on epigenetic age acceleration. Model: epigenetic age acceleration ~ each independent variable + batch information + sex + smoking status; Cardiovascular disease risk score included participants < 80 years old without cardiovascular diseases (*n* = 3982). **b** Independent effects of cardiovascular risk factors clusters on epigenetic age acceleration. Model: epigenetic age acceleration ~ lipoproteins + kidney function + inflammation + adiposity + glucose homeostasis + sex + batch information + smoking status. Abbreviations: LDL, low-density lipoproteins; HDL, high-density lipoproteins; eGFR, estimated glomerular filtration rate; CRP, C-reactive protein; BMI, body mass index; HbA1c, glycated hemoglobin; SD, standard deviation

Markers of kidney function (cystatin C and eGFR), adiposity (% body fat, BMI and waist circumference), and cardiovascular risk score were consistently associated with the four epigenetic age acceleration estimators (Fig. 2a and eTable 1). Measures of inflammation (CRP), and glucose homeostasis (insulin, insulin resistance, and blood glucose) were associated with AgeAccel.Hannum, AgeAccelPheno, and AgeAccelGrim, but not with AgeAccel.Horvath. Within the lipoprotein category, triglyceride and/or HDL levels were only associated with AgeAccelPheno and AgeAccelGrim. Moreover, compared to AgeAccel.Horvath and AgeAccel.Hannum, effect sizes of these cardiovascular risk factors were larger for AgeAccelPheno and AgeAccelGrim. Ten out of 16 cardiovascular risk factors were significantly associated with all four epigenetic age acceleration estimators when only adjusted for batch effect (eTable 1, model 1). Most of these associations remained similar for AgeAccelPheno and AgeAccelGrim upon further adjustment for sex (model 2) and smoking status (model 3), whereas effect sizes for lipoproteins and markers of glucose homeostasis changed markedly for AgeAccel.Horvath and AgeAccel.Hannum (eTable 1).

In evaluating the independent effects of the various risk factor clusters (Fig. 2b), we found that decreased kidney function, increased inflammation, and higher adiposity markers were independently associated with larger AgeAccelPheno and AgeAccelGrim. Increased inflammation and higher adiposity markers were also independently associated with larger AgeAccel.Hannum, whereas only decreased kidney function was independently associated with larger AgeAccel.Horvath.

### Relation between markers of vascular function (blood pressure, arterial stiffness, endothelial function, and hemodynamics) and epigenetic age acceleration

Hierarchical clustering of markers of vascular function yielded four categories, comprising of blood pressure (DBP, MAP, SBP), arterial stiffness (PP, TACI, PWV, ABI), endothelial function (RSH), and hemodynamics (CI, SVRI, SI) (Fig. 1b). Measures of blood pressure, arterial stiffness, endothelial function, and hemodynamics were only weakly correlated with most of the classical cardiovascular risk factors and across categories, with higher correlation within each category as expected (eFig. 1).

**Fig. 3:**
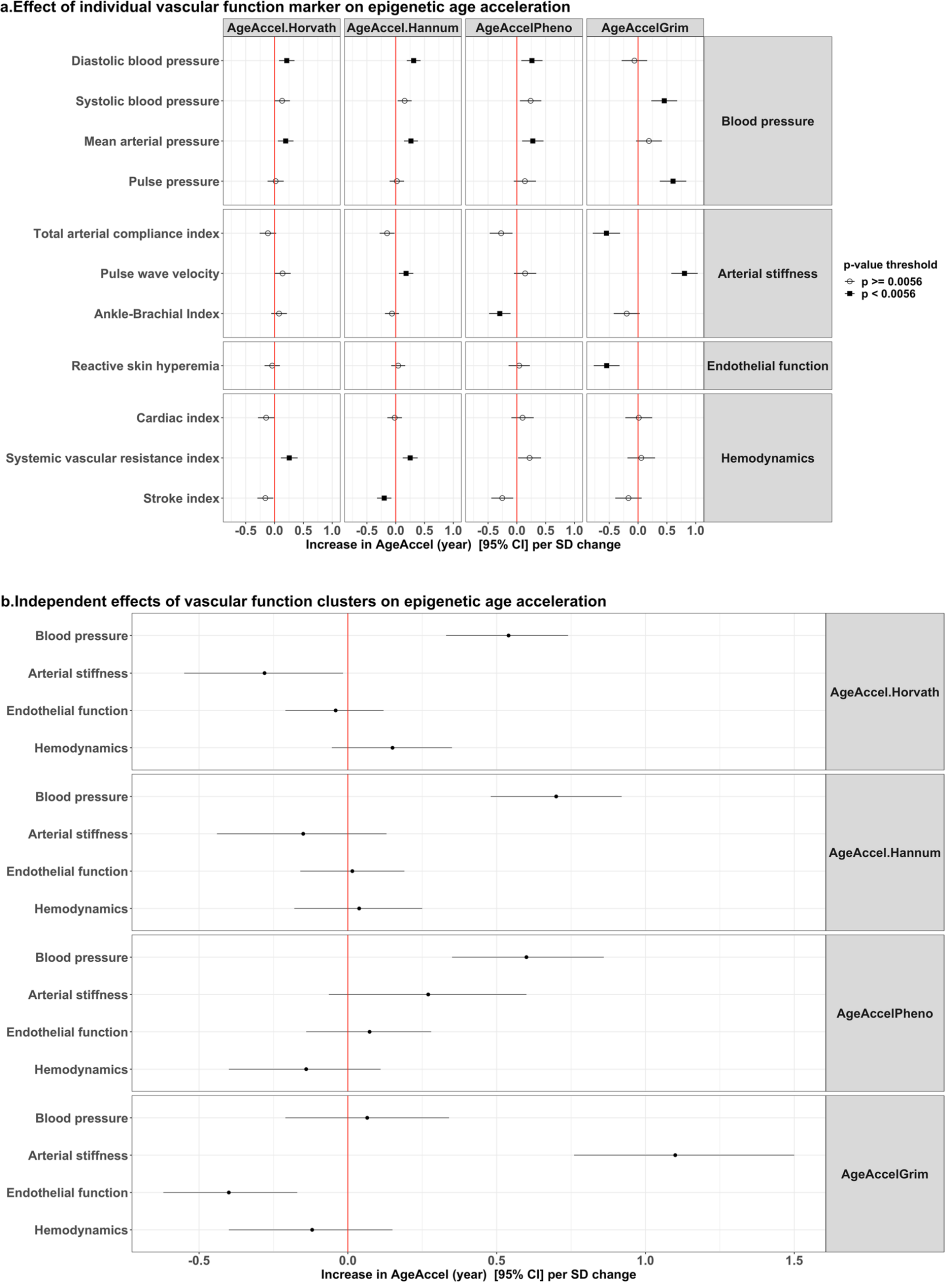
Relation between markers of vascular function and epigenetic age acceleration. **a** Effect of individual vascular function markers on epigenetic age acceleration. Model: epigenetic age acceleration ~ each independent variable + batch information + sex + smoking status. **b** Independent effects of vascular function clusters on epigenetic age acceleration. Model: epigenetic age acceleration ~ blood pressure + arterial stiffness + endothelial function + hemodynamics + sex + batch information + smoking status

When analyzed separately, higher blood pressure measures and larger arterial stiffness were consistently associated with higher epigenetic age acceleration (Fig. 3a and eTable 2). In contrast, a better hemodynamic function was associated with lower AgeAccel.Horvath and AgeAccel.Hannum, while a better endothelial function was only significantly associated with lower AgeAccelGrim.

When we evaluated the independent effects of the various vascular function clusters on epigenetic age acceleration (Fig. 3b), we found that an unfavorable blood pressure profile was associated with higher AgeAccel.Horvath, AgeAccel.Hannum, and AgeAccelPheno. Increased arterial stiffness and decreased endothelial function, however, were only associated with higher AgeAccelGrim.

### Relation between cardiovascular markers and AgeAccelGrim component variables

Except for DNAmGDF15, the DNAm-based plasma protein components of AgeAccelGrim were all associated with the classical cardiovascular risk factors (eFig. 2a). Moreover, the association between classical cardiovascular risk factors and AgeAccelGrim was mainly driven by DNAmPAI1, DNAmTIMP1, and DNAmPACKYRS. The classical cardiovascular risk factors were more strongly associated with DNAmPAI1 than with AgeAccelGrim. The association of blood pressure and arterial stiffness with AgeAccelGrim was driven by DNAmPAI1, while the association of reactive skin hyperemia and AgeAccelGrim was driven by DNAmPACKYRS (eFig. 2b).

### Sex-stratified analyses

All four epigenetic age acceleration estimators were consistently higher in men compared to women (Table 1), which is consistent with previous results [46]. The effects of classical cardiovascular risk factors on epigenetic age acceleration did not differ between men and women, except for cystatin C, eGFR, and CRP, which had a stronger association with epigenetic age acceleration in men. Regarding markers of vascular function, the effects of arterial stiffness and worse hemodynamics on epigenetic age acceleration were significantly stronger in men than in women, but similar between sexes for blood pressure traits and endothelial function (eFig. 3 and eTable 3).

### Sensitivity analyses

The estimated effects of all cardiovascular risk factors and markers of vascular function on epigenetic age acceleration remained virtually identical after exclusion of participants with diabetes or those with a history of stroke or myocardial infarction (*n* = 299). We also examined potential nonlinear relationships between cardiovascular risk factors and markers of vascular function and epigenetic age acceleration. None of the associations significantly deviated from linearity.

## Discussion

We performed a large and comprehensive study to elucidate the precise contributions of cardiovascular risk factors and quantitative markers of vascular function across multiple domains on the four most commonly used epigenetic estimators of biological aging. Through this approach, we were able to compare the utility of both first- and second-generation epigenetic age acceleration estimators in capturing multi-domain cardiovascular dysfunction. Using a population-based approach, we found that across a wide age range, individuals with an unhealthy cardiovascular risk profile, as well as those with unfavorable functional vascular parameters, consistently displayed accelerated epigenetic aging. AgeAccelPheno and AgeAccelGrim outperformed AgeAccel.Horvath and AgeAccel.Hannum in capturing multisystem dysregulation. Importantly, the effects of cardiovascular risk factors and markers of vascular function on accelerated epigenetic aging were independent, suggesting that targeting of (modifiable) cardiovascular risk profiles across different physiological domains is likely to have a cumulative effect with respect to slowing of the unhealthy aging process.

Our findings indicate that an unfavorable cardiovascular health profile could underlie interindividual differences in biological aging, contributing to unhealthy aging-related morbidity and mortality. Indeed, cardiovascular dysfunctions across multiple physiological domains were consistently associated with the four most widely used epigenetic age acceleration estimators, which capture variations in the rate of biological aging beyond chronological age. Moreover, our study also provides clues on how lifespan estimators (i.e., AgeAccelPheno and AgeAccelGrim) might capture more aspects of biological aging and outperform the first generation aging estimators (i.e., AgeAccel.Horvath and AgeAccel.Hannum) as determinants of morbidity and mortality. Aging involves complex changes across multiple physiological domains, and consequently, its pace is determined by the cumulative effects across those domains [47, 48]. Compared with Horvath and Hannum’s clocks, which do not account for the heterogeneity of physiological complexity among individuals, PhenoAge and GrimAge, include not only CpGs with strong time-dependent changes, but also those related to divergence in the rate of aging [24–26]. Indeed, we found that more cardiovascular factors with larger effect sizes were associated with AgeAccelPheno and AgeAccelGrim as compared to AgeAccel.Horvath and AgeAccel.Hannum. Collectively, these findings thus suggest that AgeAccelPheno and AgeAccelGrim more closely reflect the cumulative effects of the underlying aging-related molecular mechanisms on the epigenome [22–25, 49, 50].

Previous studies have found associations of BMI, blood pressure, and metabolic syndrome, with epigenetic age acceleration. However, these mostly focused on only a few cardiovascular risk factors or a composite cardiovascular health score [51, 52], mainly in relation to first-generation age acceleration estimators [35–37, 53]. Our findings substantially extend previous findings by showing that unfavorable changes in cardiovascular risk factors across multiple physiological domains are independently associated with the four most widely used epigenetic age acceleration estimators across a wide age spectrum. This indicates that the effects of changes in multiple cardiovascular domains are additive at the epigenetic level, suggesting an independent modification of the rate of biological aging. The relationships of many cardiovascular risk factors with increased cardiovascular-associated morbidity and decreased life expectancy have been well established [1, 42, 54]. However, the underlying molecular pathways mediating these associations are much less clear. A potential mechanism could be the influence of cardiovascular risk factors on systemic gene expression profiles through changes in DNA methylation [16, 21]. Our findings highlight a robust relation between most known cardiovascular risk factors and accelerated epigenetic aging.

Importantly, we also found that unfavorable changes in quantitative markers of vascular function were associated with accelerated epigenetic aging. Previous studies of such markers mainly focused on studying the methylation status of single genes [20, 55]. They showed that hemodynamic changes may exert part of their role in the pathogenesis of vascular diseases through epigenetic remodeling [19, 20]. As epigenetic age acceleration takes a panel of CpG changes into account, our findings support the notion that cardiovascular dysfunction may induce multiple methylation changes across the epigenome, which could have an impact on the rate of biological aging. We also found that endothelial function was associated with AgeAccelGrim, suggesting that this lifespan estimator may be able to capture the molecular signature of endothelial dysfunction associated with CVDs. Although the precise molecular mechanisms remain to be elucidated, this may occur through loss of proteostasis involving vascular remodeling, inflammation, and immune dysfunction: Plasma proteins used in the construction of GrimAge, including adrenomedullin [56], beta-2- macroglobulin [57], growth differentiation factor 15 [58], and plasminogen activator inhibitor 1 [59], are markers of inflammation response and immune function which have been linked to CVDs.

Our study has both strengths and limitations. First, we were able to scrutinize the effects of a wide range of cardiovascular risk factors and quantitative markers of vascular function conjointly in a large study concerning the relation between cardiovascular and accelerated epigenetic aging. Second, we present results for the four most widely used epigenetic age acceleration estimators, showing that although the effects are consistent across the different estimators, AgeAccelPheno and AgeAccelGrim more closely reflect changes in cardiovascular risk factors. Third, our estimates are based on a broad age spectrum, ranging from 30 to 95 years old, and are therefore likely to represent the association between cardiovascular and accelerated epigenetic aging across most of the adult lifespan. On the other hand, the cross-sectional nature of our study precludes formal evaluation of the directionality of the effects. So, although we consider it likely, based on findings from prior studies showing that cardiovascular risk factors trigger changes in DNA methylation [12–20, 60], the converse cannot be excluded. Another limitation of our study could be that we defined participants’ diabetes and hypertension status based on self-reports in conjunction with current regular use of anti-diabetic and anti-hypertensive medications, respectively, which could potentially have introduced a (non-differential) misclassification bias; however, if anything, this is likely to have biased the results towards the null.

## Conclusion

In conclusion, we found that multiple cardiovascular risk factors and quantitative markers of vascular function across different physiological systems were consistently and independently associated with accelerated epigenetic aging. Therefore, promoting cardiovascular health may lower epigenetic age acceleration, with potential health impacts that go beyond the purely cardiovascular aspects of the aging process.

## Supplementary Information

Below is the link to the electronic supplementary material.

Supplementary file1 (DOCX 12.3 MB)

Supplementary file2 (XLSX 24 KB)

## Data Availability

The Rhineland Study’s dataset is not publicly available because of data protection regulations. Access to data can be provided to scientists in accordance with the Rhineland Study’s Data Use and Access Policy. Requests for further information or to access the Rhineland Study’s dataset should be directed to RS-DUAC@dzne.de. All the authors had full access to all the data in the study, and the corresponding author takes responsibility for data integrity.

## References

[1] Collaborators, G.B.D.C.o.D., Global, regional, and national age-sex specific mortality for 264 causes of death, 1980–2016: a systematic analysis for the Global Burden of Disease Study 2016. Lancet. 2017;390(10100): p. 1151–1210. 10.1016/S0140-6736(17)32152-9 PMC5605883 28919116

[2] North BJ, Sinclair DA (2012). The intersection between aging and cardiovascular disease. Circ Res.

[3] Ben-Shlomo Y (2014). Aortic pulse wave velocity improves cardiovascular event prediction: an individual participant meta-analysis of prospective observational data from 17,635 subjects. J Am Coll Cardiol.

[4] Medina-Lezama J (2018). Hemodynamic patterns identified by impedance cardiography predict mortality in the general population: the PREVENCION study. J Am Heart Assoc.

[5] Patel SA (2015). Cardiovascular mortality associated with 5 leading risk factors: national and state preventable fractions estimated from survey data. Ann Intern Med.

[6] Yusuf S (2020). Modifiable risk factors, cardiovascular disease, and mortality in 155 722 individuals from 21 high-income, middle-income, and low-income countries (PURE): a prospective cohort study. Lancet.

[7] Hamczyk MR (2020). Biological versus chronological aging: JACC focus seminar. J Am Coll Cardiol.

[8] Montiel Rojas D (2018). Short telomere length is related to limitations in physical function in elderly European adults. Front Physiol.

[9] Bell JT (2012). Epigenome-wide scans identify differentially methylated regions for age and age-related phenotypes in a healthy ageing population. PLoS Genet.

[10] Fraga MF, Esteller M (2007). Epigenetics and aging: the targets and the marks. Trends Genet.

[11] Jones MJ, Goodman SJ, Kobor MS (2015). DNA methylation and healthy human aging. Aging Cell.

[12] Aref-Eshghi E (2020). Glucose-induced, duration-dependent genome-wide DNA methylation changes in human endothelial cells. Am J Physiol Cell Physiol.

[13] Sun D (2019). Body mass index drives changes in DNA methylation: a longitudinal study. Circ Res.

[14] Wahl S (2017). Epigenome-wide association study of body mass index, and the adverse outcomes of adiposity. Nature.

[15] Mudry JM (2017). Insulin and glucose alter death-associated protein kinase 3 (DAPK3) DNA methylation in human skeletal muscle. Diabetes.

[16] Mendelson MM (2017). Association of body mass index with DNA methylation and gene expression in blood cells and relations to cardiometabolic disease: a mendelian randomization approach. PLoS Med.

[17] Dekkers KF (2016). Blood lipids influence DNA methylation in circulating cells. Genome Biol.

[18] Horvath S (2014). Obesity accelerates epigenetic aging of human liver. Proc Natl Acad Sci U S A.

[19] Dunn J (2014). Flow-dependent epigenetic DNA methylation regulates endothelial gene expression and atherosclerosis. J Clin Invest.

[20] Jiang YZ (2014). Hemodynamic disturbed flow induces differential DNA methylation of endothelial Kruppel-like factor 4 promoter in vitro and in vivo. Circ Res.

[21] Richard MA (2017). DNA methylation analysis identifies loci for blood pressure regulation. Am J Hum Genet.

[22] Hannum G (2013). Genome-wide methylation profiles reveal quantitative views of human aging rates. Mol Cell.

[23] Horvath S (2013). DNA methylation age of human tissues and cell types. Genome Biol.

[24] Levine ME (2018). An epigenetic biomarker of aging for lifespan and healthspan. Aging (Albany NY).

[25] Lu AT (2019). DNA methylation GrimAge strongly predicts lifespan and healthspan. Aging (Albany NY).

[26] Horvath S, Raj K (2018). DNA methylation-based biomarkers and the epigenetic clock theory of ageing. Nat Rev Genet.

[27] Breitling LP (2016). Frailty is associated with the epigenetic clock but not with telomere length in a German cohort. Clin Epigenetics.

[28] Chen BH (2016). DNA methylation-based measures of biological age: meta-analysis predicting time to death. Aging (Albany NY).

[29] Marioni RE (2015). DNA methylation age of blood predicts all-cause mortality in later life. Genome Biol.

[30] Marioni RE (2015). The epigenetic clock is correlated with physical and cognitive fitness in the Lothian Birth Cohort 1936. Int J Epidemiol.

[31] Roetker NS (2018). Prospective study of epigenetic age acceleration and incidence of cardiovascular disease outcomes in the ARIC study (Atherosclerosis Risk in Communities). Circ Genom Precis Med.

[32] Fransquet PD (2019). The epigenetic clock as a predictor of disease and mortality risk: a systematic review and meta-analysis. Clin Epigenetics.

[33] McCrory C (2021). GrimAge outperforms other epigenetic clocks in the prediction of age-related clinical phenotypes and all-cause mortality. J Gerontol A Biol Sci Med Sci.

[34] Protsenko E (2021). "GrimAge," an epigenetic predictor of mortality, is accelerated in major depressive disorder. Transl Psychiatry.

[35] Huang RC (2019). Epigenetic age acceleration in adolescence associates with BMI, inflammation, and risk score for Middle Age cardiovascular disease. J Clin Endocrinol Metab.

[36] Quach A (2017). Epigenetic clock analysis of diet, exercise, education, and lifestyle factors. Aging (Albany NY).

[37] Nannini DR (2019). Epigenetic age acceleration and metabolic syndrome in the coronary artery risk development in young adults study. Clin Epigenetics.

[38] Fortin JP, Triche TJ, Hansen KD (2017). Preprocessing, normalization and integration of the Illumina HumanMethylationEPIC array with minfi. Bioinformatics.

[39] Wu MC, Kuan PF (2018). A guide to Illumina BeadChip data analysis. Methods Mol Biol.

[40] Inker LA (2012). Estimating glomerular filtration rate from serum creatinine and cystatin C. N Engl J Med.

[41] Collerton J (2007). Telomere length is associated with left ventricular function in the oldest old: the Newcastle 85+ study. Eur Heart J.

[42] D'Agostino RB (2008). General cardiovascular risk profile for use in primary care: the Framingham Heart Study. Circulation.

[43] Mapstone M (2014). Plasma phospholipids identify antecedent memory impairment in older adults. Nat Med.

[44] Aboyans V (2012). Measurement and interpretation of the ankle-brachial index: a scientific statement from the American Heart Association. Circulation.

[45] Choi PJ, et al. New approach to measure cutaneous microvascular function: an improved test of NO-mediated vasodilation by thermal hyperemia. J Appl Physiol (1985). 2014;117(3):277–83 10.1152/japplphysiol.01397.2013 PMC4122693 24903917

[46] Horvath S (2016). An epigenetic clock analysis of race/ethnicity, sex, and coronary heart disease. Genome Biol.

[47] Harman D (1991). The aging process: major risk factor for disease and death. Proc Natl Acad Sci U S A.

[48] Khan SS, Singer BD, Vaughan DE (2017). Molecular and physiological manifestations and measurement of aging in humans. Aging Cell.

[49] Schottker B (2020). Oxidatively damaged DNA/RNA and 8-isoprostane levels are associated with the development of type 2 diabetes at older age: results from a large cohort study. Diabetes Care.

[50] Gao X (2019). The associations of DNA methylation alterations in oxidative stress-related genes with cancer incidence and mortality outcomes: a population-based cohort study. Clin Epigenetics.

[51] Lo YH, Lin WY (2022). Cardiovascular health and four epigenetic clocks. Clin Epigenetics.

[52] Pottinger TD (2021). Association of cardiovascular health and epigenetic age acceleration. Clin Epigenetics.

[53] Nevalainen T (2017). Obesity accelerates epigenetic aging in middle-aged but not in elderly individuals. Clin Epigenetics.

[54] Yusuf S (2004). Effect of potentially modifiable risk factors associated with myocardial infarction in 52 countries (the INTERHEART study): case-control study. Lancet.

[55] Murray R (2016). DNA methylation at birth within the promoter of ANRIL predicts markers of cardiovascular risk at 9 years. Clin Epigenetics.

[56] Kita T, Kitamura K (2022). Translational studies of adrenomedullin and related peptides regarding cardiovascular diseases. Hypertens Res.

[57] Shi F, Sun L, Kaptoge S (2021). Association of beta-2-microglobulin and cardiovascular events and mortality: a systematic review and meta-analysis. Atherosclerosis.

[58] Wang J (2019). Roles of growth differentiation factor 15 in atherosclerosis and coronary artery disease. J Am Heart Assoc.

[59] Tofler GH (2016). Plasminogen activator inhibitor and the risk of cardiovascular disease: the Framingham heart study. Thromb Res.

[60] Joyce BT (2021). Epigenetic age acceleration reflects long-term cardiovascular health. Circ Res.

