# Supplementary material for: Cardiovascular correlates of epigenetic aging across the adult lifespan: a population-based study
Source: GeroScience. 2023 Feb 8;45(3):1605–18. doi: 10.1007/s11357-022-00714-0 (PMC10400487; doi:10.1007/s11357-022-00714-0)
Supplement: Supplementary file 1 — Supplementary file1 (DOCX 12.3 MB) [file 11357_2022_714_MOESM1_ESM.docx]

**Cardiovascular Correlates of Epigenetic Aging across the Adult Lifespan: A Population-based Study**

**Authors**

Dan Liu, PhD^1^, N. Ahmad Aziz, MD PhD^1,2^, Gökhan Pehlivan MD^1^, Monique M.B. Breteler, MD PhD^1,3*^

***Corresponding Author:**

Monique M.B. Breteler, MD PhD

Director of Population Health Sciences

German Center for Neurodegenerative diseases (DZNE)

Venusberg-Campus 1, Building 99

53127 Bonn

Germany

Tel +49 228 43302-929

Email: [monique.breteler@dzne.de](mailto:monique.breteler@dzne.de)

ORCID: https://orcid.org/**0000-0002-0626-9305**

**Supplemental Material**

eFig 1. Correlation structure of cardiovascular factors

eFig 2. Relation between cardiovascular markers and AgeAcceGrim component variables

eFig 3. Assessment of interaction effects between sex and cardiovascular factors

eTable 1. Effects of classical cardiovascular risk factors on epigenetic age acceleration

eTable 2. Effects of markers of vascular function on epigenetic age acceleration

eTable 3. Sex-stratified analyses of the relation between cardiovascular factors and epigenetic age acceleration

**eFig 1. Correlation structure of cardiovascular factors**


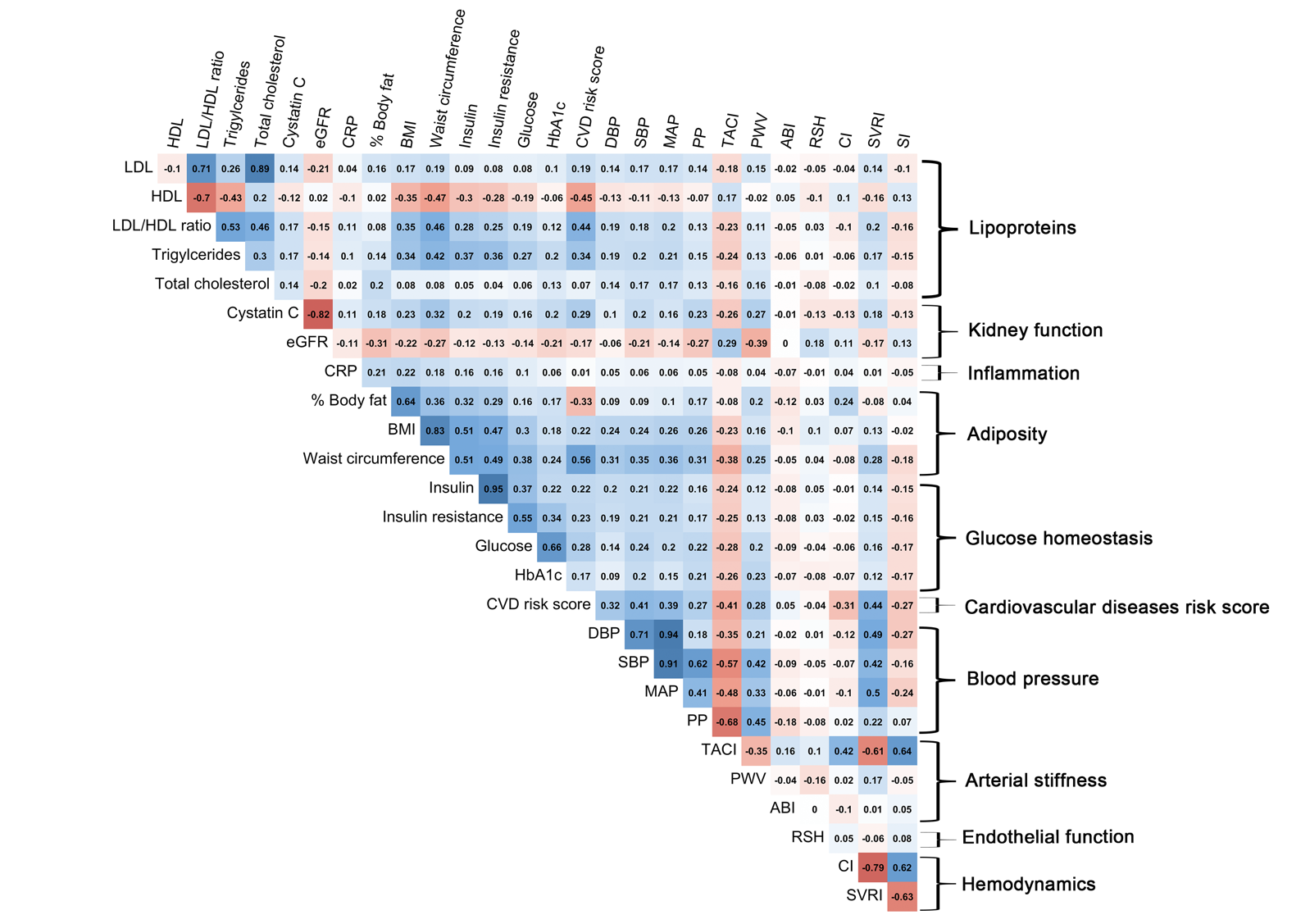


Abbreviations: LDL, low-density lipoproteins; HDL, high-density lipoproteins; eGFR, estimated glomerular filtration rate; CRP, C-reactive protein; BMI, body mass index; HbA1c, glycated hemoglobin; CVD, cardiovascular diseases; DBP, diastolic blood pressure; SBP, systolic blood pressure; MAP, mean arterial pressure; PP, pulse pressure; TACI, total arterial compliance index; PWV, pulse wave velocity; ABI, ankle-brachial index; RSH, reactive skin hyperemia; CI, cardiac index; SVRI, systemic vascular resistance index; SI, stroke index.

**eFig 2. Relation between cardiovascular markers and AgeAcceGrim component variables**

**a. classical cardiovascular risk factors**

**
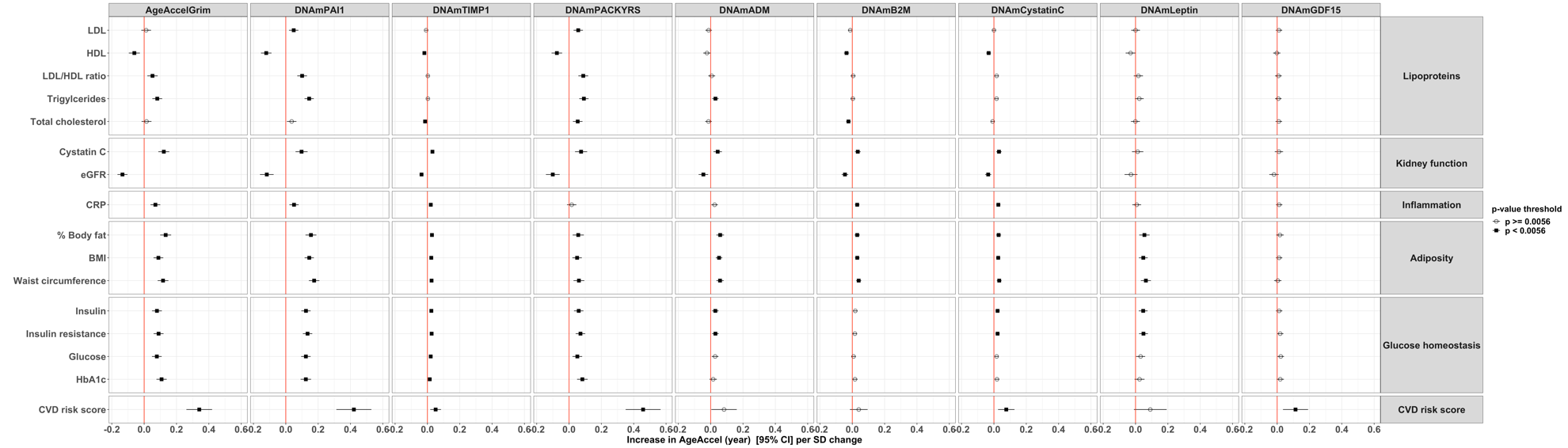
**

**b. markers of vascular function**

**
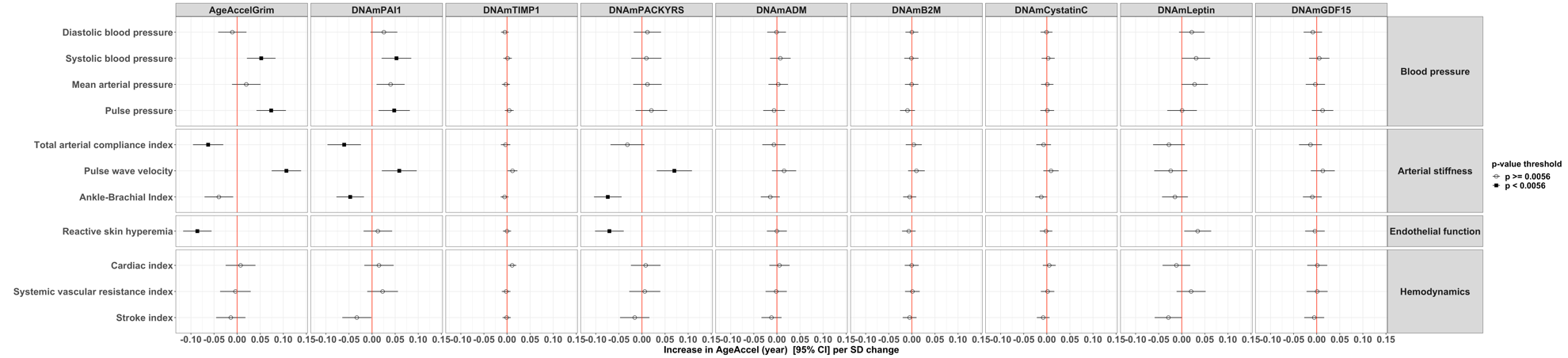
**

Abbreviations: DNAm: DNA methylation; ADM, adrenomedullin; B2M, beta‐2 microglobulin; GDF-15, growth differentiation factor 15; PAI-1, plasminogen activation inhibitor 1; TIMP-1, tissue inhibitor metalloproteinase 1; PACKYRS, smoking pack-years; LDL, low-density lipoproteins; HDL, high-density lipoproteins; eGFR, estimated glomerular filtration rate; CRP, C-reactive protein; BMI, body mass index; HbA1c, glycated hemoglobin; SD, standard deviation. Cardiovascular disease risk score included participants < 80 years old without cardiovascular diseases (n=3982).

**eFig 3. Assessment of interaction effects between sex and cardiovascular factors on epigenetic age acceleration**

**
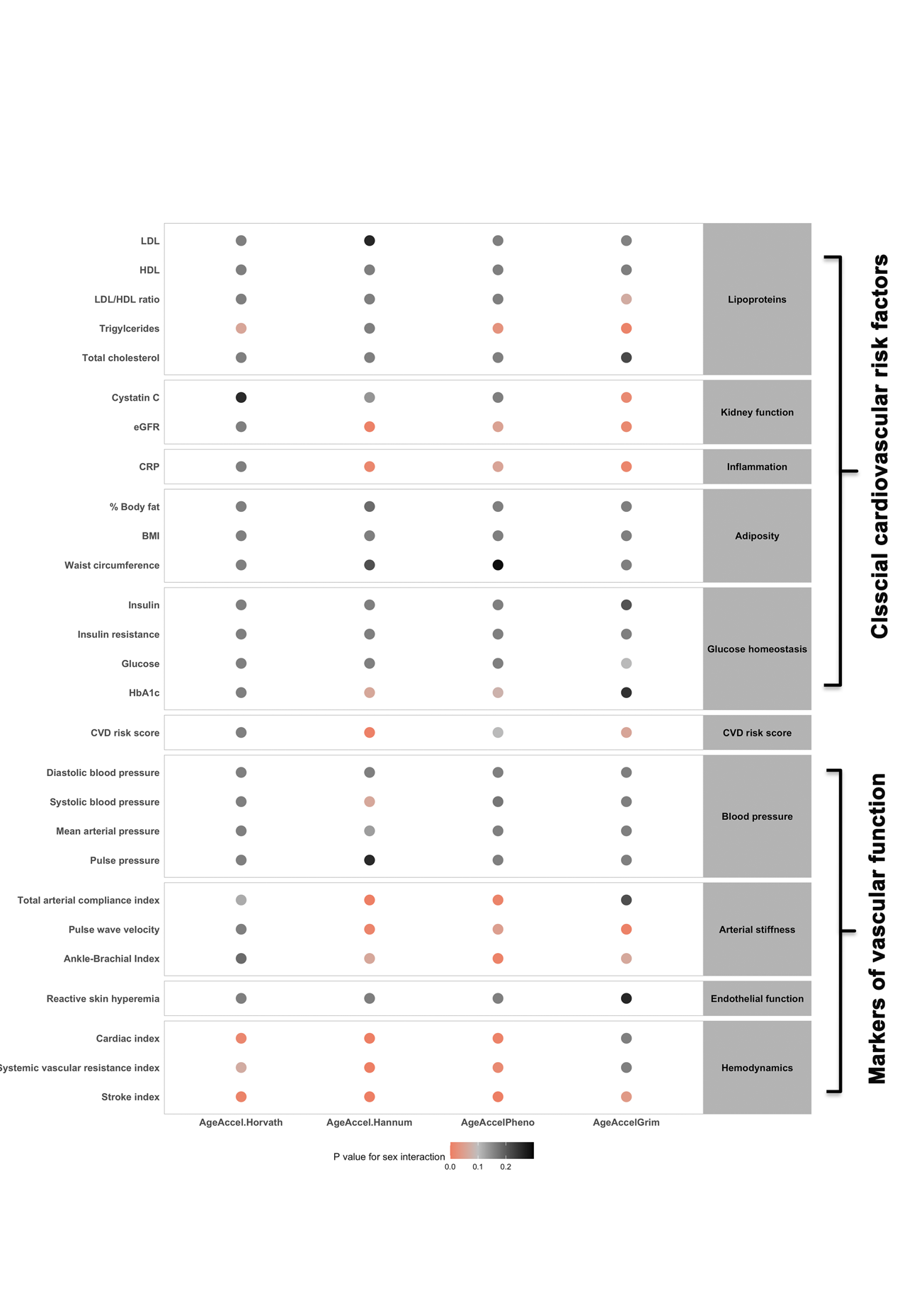
**

Abbreviations: LDL, low-density lipoproteins; HDL, high-density lipoproteins; eGFR, estimated glomerular filtration rate; CRP, C-reactive protein; BMI, body mass index; HbA1c, glycated hemoglobin; CVD, cardiovascular diseases; DBP, diastolic blood pressure; SBP, systolic blood pressure; MAP, mean arterial pressure; PP, pulse pressure.
